# Fiery Rash Revealing Hidden Hemochromatosis: A Case Study

**DOI:** 10.7759/cureus.95896

**Published:** 2025-11-01

**Authors:** Annabel Crippen, Anushka Parekh, Ruiling Yuan

**Affiliations:** 1 Medicine, Edward Via College of Osteopathic Medicine, Spartanburg, USA; 2 Hematology and Oncology, Self Regional Medical Center, Greenwood, USA

**Keywords:** atypical rash, dermatologic manifestations, hereditary hemochromatosis (hh), iron overload, juvenile

## Abstract

Hereditary hemochromatosis (HH) is an autosomal recessive genetic disorder characterized by excessive iron accumulation in tissues. This case highlights a 29-year-old man with an unusual presentation of hemochromatosis. Persistent symptoms led to bloodwork that revealed iron overload, prompting further genetic analysis.

The 29-year-old Caucasian man presented to his primary care provider (PCP) with complaints of an erythematous and pruritic rash across his chest and abdomen. It was thought to be irritant contact dermatitis in congruence with his past medical history. He was treated with steroids with temporary improvement. He was also referred to dermatology, where he was started on dupilumab injections for eczema. Two weeks later, the patient visited the emergency department, presenting with an erythematous and pustular rash diffusely throughout both feet. His rash was assumed to be eczematous, and he was given steroids and antibiotics due to a few open sores observed. Upon returning to his PCP for follow-up, his routine labs revealed an elevated iron level of 208. In conjunction with his skin irritation, a cheek swab was ordered to check for the C282Y gene. This prompted his new diagnosis of HH.

Upon this result, he was referred to hematology for further work-up. Magnetic resonance imaging (MRI) of the abdomen showed evidence of diffuse iron deposition in the liver. An echocardiogram and thyroid-stimulating hormone (TSH) levels were found to be normal in this patient. Treatment was explained, and he consented to start weekly phlebotomy to reduce total body iron stores.

This case highlights an initial presentation that deviates from the typical rash and age group seen in hemochromatosis. Recognizing such manifestations may facilitate earlier diagnosis, and physicians should consider iron studies in patients with eczema or irritant dermatitis that is resistant or persistent despite standard therapies.

## Introduction

Hereditary hemochromatosis (HH) is a genetic disorder in which the body absorbs and stores excess iron, resulting in iron overload and the deposition of iron throughout the body. HH is the most common autosomal recessive genetic disorder in people of Northern European descent. In the United States, the disease prevalence is one case per 200-400 people [1]. Hemochromatosis presents more frequently in men, typically between the fourth and sixth decades. Women, on the other hand, are often diagnosed later due to iron loss through menstruation [2]. The most common cause is a homozygous C282Y mutation in the HFE gene. Less frequent is a compound mutation of p.C282Y and p.H63D in the HFE gene, often leading to a less severe phenotype [3]. The mutation in the HFE gene results in the dysregulation of the protein hepcidin. Hepcidin is therefore no longer able to regulate iron levels in the body, causing an increase in iron uptake from the diet and iron release from macrophages and hepatocytes. Without proper regulation from functional hepcidin proteins, continuous iron absorption can result in iron overload [4]. Alternatively, hemochromatosis may present secondary to chronic blood transfusions in patients with thalassemia, sickle cell disease, and hereditary spherocytosis [2]. Chronic blood transfusion therapies introduce an excessive amount of iron that is absorbed and stored in organs such as the heart, liver, and pancreas [5]. It is important to consider secondary causes when investigating possible hemochromatosis. HH is typically diagnosed with genetic testing following an abnormal iron panel or family history of hemochromatosis.

Excess iron deposits are found in organs such as the liver, pancreas, heart, joints, skin, and pituitary gland, leading to the dysfunction of many bodily systems. Although most patients are asymptomatic, iron deposition across organ systems leads to a wide array of possible presentations such as hypothyroidism, hypopituitarism, fatigue, arrhythmias, cardiomyopathy, arthralgias, abdominal pain, hepatomegaly, jaundice, xerosis, and amenorrhea [6]. The classic triad of hemochromatosis is skin hyperpigmentation, cirrhosis, and diabetes mellitus [4]. This presentation contributes to the popular moniker given to hemochromatosis of "bronze diabetes". This term, however, describes late findings in the course of this disease, and patients are at risk for liver cirrhosis, hepatocellular carcinoma, and hepatic encephalopathy [6]. It is imperative that a low threshold is held for hemochromatosis investigation, especially due to vague early symptoms such as arthralgias and fatigue [6]. 

Dermatologic manifestations have yet to be thoroughly studied in their various presentations [7]. This unique case highlights a 29-year-old man with an unusual presentation of HH. Persistent symptoms of dermatitis led to obtaining labs that revealed iron overload, prompting further genetic analysis.

## Case presentation

A 29-year-old Caucasian man with no significant medical history presented to his primary care provider (PCP) with complaints of fatigue and an erythematous, macular, pruritic rash. He reported that this rash had been present across his chest and abdomen persistently for three months. It was initially attributed to irritant contact dermatitis, given that his rash was on his abdomen, near a nickel belt buckle. His frequent history of urgent care visits in 2018 also corroborated this diagnosis. Fatigue preceded this rash, and he also endorsed mild arthralgias. He denied any fever or weight loss. He was treated with a 4 mg methylprednisolone course and a dexamethasone sodium phosphate 4 mg/mL injection. The lack of alleviated symptoms led to the patient being referred to dermatology. He was diagnosed with atopic dermatitis and started receiving monthly dupilumab injections. Despite this, the erythematous, macular, pruritic rash on his chest and abdomen persisted. This persistence, along with his history, led to a presumed irritant dermatitis etiology.

**Figure 1:**
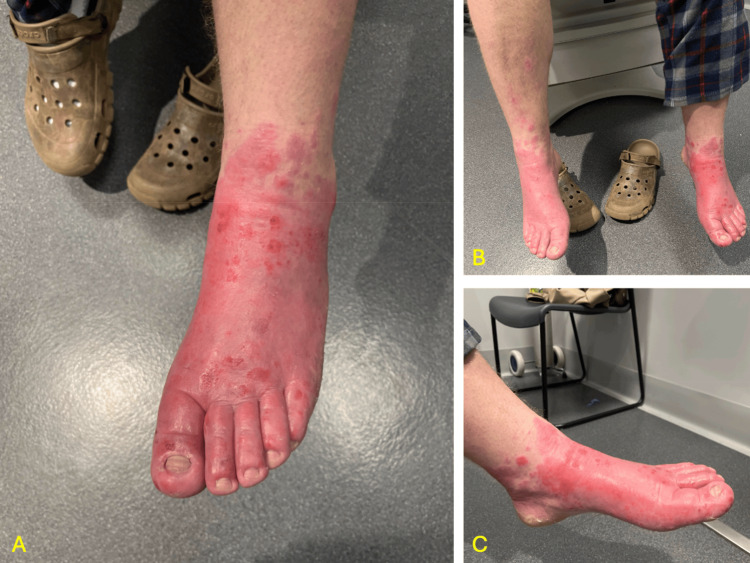
Images of novel rash across both feet (A) Anterior view of the left foot showing a diffuse erythematous rash with numerous pustules. (B) Wide anterior view of both feet demonstrating a diffuse erythematous rash with multiple pustules. (C) Medial view of the left foot highlighting the diffuse erythematous rash.

Two weeks later, the patient visited the emergency department, presenting with a more extensive rash. The rash from his chest and abdomen had alleviated, but a new rash featuring an erythematous base with numerous pustules diffusely across the dorsum of both feet was present. This is shown in Figure 1. Due to recent contact exposure to new laundry detergent and sawdust, his rash was again assumed to be irritant. The patient had been using 1%-0.05% clotrimazole-betamethasone cream prior to presenting at the emergency department, but it resulted in skin peeling. The patient was given an intramuscular dexamethasone injection and a course of oral cephalexin for a few open sores observed, which were concerning for a secondary bacterial infection. After no change in symptoms, his PCP ordered routine labs to explore alternative causes given his fatigue. These new labs revealed an abnormal iron panel, shown in Table 1. The elevated ferritin led the provider to order a cheek swab to check for the C282Y gene. The finding of a homozygous mutation in the HFE gene confirmed his new diagnosis of HH. 

**Table 1:** Iron studies from the initial primary care provider visit and recent hematology visit

Iron panel	Patient values (11/12/2024)	Patient values (02/10/2025)	Reference range
Serum iron	208 mcg/dL	108 mcg/dL	60-170 mcg/dL
Ferritin	719 ng/mL	473.4 ng/mL	24-336 ng/mL
Transferrin saturation	75%	40%	20-50%
Total iron binding capacity	279 mcg/dL	269 mcg/dL	250-450 mcg/dL
Transferrin	-	192 mg/dL	215-380 mg/dL

**Figure 2:**
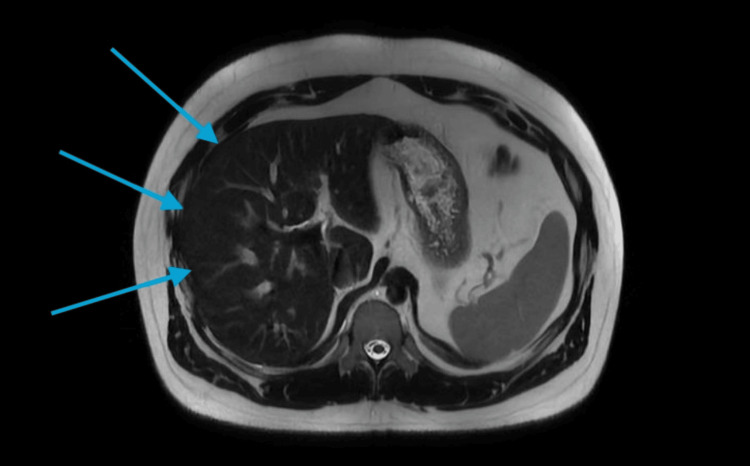
Magnetic resonance imaging showing diffuse signal loss on T2 imaging (arrows) throughout the liver which is present with evidence of signal increase on opposed gradient echo imaging indicating diffuse sign infiltration

Upon this result, he was referred to hematology for further work-up. At the hematology office, routine labs such as complete blood count, iron panel, and complete metabolic panel were ordered again to obtain his baseline. The iron panel was once again abnormal, with the patient's values shown in Table 1. A magnetic resonance imaging (MRI) and an echocardiogram were ordered to identify iron deposition in the body. Figure 2 shows the MRI of the abdomen demonstrating diffuse signal loss on T2 imaging consistent with diffuse iron deposition in the liver.

The echocardiogram showed an ejection fraction within normal limits (55-65%) with no evidence of structural abnormalities. The thyroid-stimulating hormone (TSH) levels were found to be normal in this patient. Treatment options were explained to the patient, who agreed to start weekly phlebotomy to reduce total body iron stores. He was also counseled on dietary modifications, including avoidance of supplements containing vitamin C and iron, as well as red meat and alcohol [1]. 

The patient was concerned for his child, given that he has no known family history of HH. It was recommended that his child should be genetically tested once he has reached adulthood. 

## Discussion

A unique characteristic of this case is the patient's age. The median age of onset in men with hemochromatosis is 40-60 years old. The most common genetic mutation in patients with HH, also known as type 1 hemochromatosis, is in the HFE gene [2]. Our patient was diagnosed with type 1 HH at the age of 29, and he had recurrent cutaneous symptoms for several years prior to diagnosis. A thought was that this patient may have had juvenile hemochromatosis. However, our patient had characteristics of classic adult-onset hemochromatosis, with his slow progressive onset of vague fatigue, arthralgia, and rashes. The unremarkable cardiac findings on echocardiogram support this. Juvenile HH, on the other hand, is typically a rapid-onset disease process presenting earlier in life with severe features such as cardiomyopathy, hypogonadism, and cirrhosis [8]. 

Early diagnosis can help prevent morbidity from all types of hemochromatosis. A study showed that one in five men and one in 10 women develop morbidity associated with hemochromatosis. In another study with HFE homozygotes, the average age of diagnosis was 61 for men and 58.5 for women, representing a systematic failure to diagnose [9]. This provides evidence that hemochromatosis may be difficult to diagnose, as it often presents asymptomatically or as nonspecific symptoms in its early stages. Literature supports the diagnosis of hemochromatosis when serum ferritin is greater than 200 µg/L in women or 300 μg/L in men or transferrin saturation >45% [2]. Clinicians often rely on family history or iron studies to diagnose HH; however, elevated ferritin levels are not always specific [6]. Other common conditions, such as alcohol use, metabolic syndrome, and fatty liver, can also contribute to elevated ferritin. Early detection methods, such as an iron panel, are inexpensive and easy to access, and there should be a low threshold to test patients who have potential signs of iron overload. After an initial abnormal iron panel, genetic testing can be done as it remains the gold standard for diagnosis [9]. 

The inherent pathophysiology of HH is well understood; however, the wide array of clinical presentations creates a barrier to early detection and treatment. In particular, dermatologic manifestations of hemochromatosis have not been thoroughly investigated [7]. The most common cutaneous presentation is hyperpigmentation, hypothesized to be due to either hemosiderin deposition in the basal cell layer of skin or an increase in melanin [10]. Unexplained pruritus resistant to common treatment is another common skin finding, as well as ichthyosis, greyish skin, hypertrichosis, and atrophic scars [7]. Other presentations to note were generalized pruritus, alopecia, and papulonodular lesions on the dorsum of the hands. Hypertrichosis and skin atrophy typically present in sun-exposed areas such as the dorsum of the hands. This is in contrast to the blistering red rash present on our patient's lower extremities, abdomen, and chest [7]. This nonspecific rash masquerading as recurrent dermatitis fostered a delay in diagnosis and treatment due to the indistinct presentation. 

The patient has been adhering to his phlebotomy regimen but has not followed up with the hematology provider since being diagnosed; therefore, it is unclear whether his skin findings have improved with treatment. Phlebotomy typically improves skin hyperpigmentation, but given this patient's atypical rash, it is unclear whether reducing iron load would fully reverse the damage. A study found that a patient's ichthyotic findings were not alleviated with phlebotomy, which could suggest that, in atypical skin findings, there may be an alternative underlying pathophysiology [11]. It is unclear whether the abundance of iron causes an immune response, such as irritant dermatitis, or directly disrupts the skin barrier [12]. This further explains the uncertainty in phlebotomy being a definitive treatment for atypical dermatologic presentations. This highlights the gap in the literature in both the pathophysiology of dermatologic manifestations and the forms of treatment offered to patients.

## Conclusions

This case highlights an initial presentation that deviates from the typical findings and age group seen in HFE-associated hemochromatosis. Recognizing such manifestations may facilitate earlier diagnosis and treatment, potentially preventing long-term complications such as liver cirrhosis, diabetes mellitus, and dilated cardiomyopathy. Since treatment is accessible and effective, providers should have a lower threshold to consider iron studies in patients with resistant dermatitis that persists despite standard therapies.
